# A cohort study of routinely used sepsis biomarkers and 28-day mortality

**DOI:** 10.1186/cc11966

**Published:** 2013-03-19

**Authors:** M De La Torre-Prados, A Garcia-De la Torre, C Trujillano-Férnández

**Affiliations:** 1 Hospital Virgen de la Victoria, Málaga, Spain; 2 Hospital Puerto Real, Cádiz, Spain

## Introduction

The evaluation of sepsis severity is complicated by the highly variable and nonspecific nature of clinical signs and symptoms. We studied routinely used biomarkers together with clinical parameters to compare their prognostic value for severe sepsis and evaluate their usefulness. Methods

A cohort study of 150 patients >18 years with severe sepsis according to the Surviving Sepsis Campaign, in an ICU of a university hospital. Demographic, clinical parameters and coagulation, infection and inflammation parameters during the first 24 hours from severe sepsis or septic shock onset were studied. Descriptive and comparative statistical analysis was performed using SPSS version 15.0 (SPSS Inc., Chicago, IL, USA).

## Results

We analyzed 150 consecutive episodes of severe sepsis (16%) or septic shock (84%) in the ICU. The median age of the patients was 64 (interquartile range, 48.7 to 71) years; the main sources of infection were intra-abdomen (45%) and respiratory (38%); 70.7% had medical diseases. The 28-day mortality was 22.7%. The profile of death patients were men (64.7%, *n *=22), with significantly higher average age (63 vs. 57 years; *P *= 0.049), as well as clinical severity scores, APACHE II (29.8 vs. 24.1; *P <*0.001) and SOFA (12.1 vs. 8.9; *P <*0.001) and major dysfunction organ number (4.6 vs. 3.6; *P <*0.001). Bilirubin was the best predictor of 28-day mortality with the largest AUC (0.71), followed by hemoglobin (0.69) and C3 (0.67). The multivariate logistic regression was adjusted for three risk parameters, hemoglobin (OR: 0.68; 95% CI: 0.51 to 0.94), bilirubin (OR:1.63; 95% CI: 1.08 to 2.45) and white blood cells (OR:1.04; 95% CI: 1.01 to 1.08) and with these parameters a ROC analysis was performed, giving an AUC of 0.77 (0.69 to 0.84).

## Conclusion

The assessment of routine biomarkers (bilirubin, white blood cells and hemoglobin) may be a helpful tool in the decision-making process at the bedside, for the evaluation of early ICU admission of recoverable patients, as indicators of inflammatory response, organ dysfunction or catabolism level, and their significant predictive value on mortality.
